# Digitally fabricated custom esthetic crowns for primary molars: A systematic review of in vitro and clinical evidence

**DOI:** 10.4317/jced.64190

**Published:** 2026-07-29

**Authors:** Mahrad Moradi, Mahtab Moradi, Maria Cruz Lorenzo-Luengo, Adrián Curto-Aguilera

**Affiliations:** 1 Department of Surgery, Faculty of Medicine, University of Salamanca, Salamanca, Spain; 2 University of the Basque Country (UPV/EHU), Leioa, Spain

## Abstract

**Background:**

The use of digitally fabricated esthetic crowns for restoring primary molars has increased with the development of CAD/CAM systems, three-dimensional (3D) printing, and tooth-colored biomaterials. However, the available evidence remains heterogeneous, and the clinical role of customized digital crowns in comparison with conventional pediatric crowns is still unclear. This systematic review aimed to assess the available in vitro and clinical evidence on esthetic customized crowns fabricated using digital workflows for primary molars.

**Material and Methods:**

A systematic literature search was conducted in PubMed, Scopus, and Web of Science to identify studies evaluating digitally fabricated esthetic crowns for primary molars. Eligible studies included in vitro investigations, clinical studies, randomized clinical trials, and finite element analyses assessing marginal or internal adaptation, fracture resistance, wear behavior, clinical performance, gingival health, or patient and parent satisfaction. Risk of bias was assessed using RoB 2 for clinical trials and the QUIN tool for in vitro studies. Due to heterogeneity in study designs, materials, comparators, and outcomes, a qualitative synthesis was performed.

**Results:**

Twenty-four studies were included, 17 in vitro studies, six clinical studies, and one finite element analysis. Most studies evaluated zirconia, 3D-printed resins, PMMA, CAD/CAM composites, or hybrid ceramics. In vitro findings suggested favorable marginal and internal adaptation for several customized digital crowns and material-dependent fracture resistance. Clinical studies reported acceptable short-term performance, although follow-up was generally limited and outcomes varied across materials. Stainless steel crowns remained a highly predictable comparator, while esthetic digital crowns showed potential advantages in esthetics and customization.

**Conclusions:**

Digitally fabricated esthetic crowns for primary molars represent a promising restorative alternative, but current evidence does not demonstrate consistent superiority over stainless steel crowns. Further well-designed clinical trials with longer follow-up and standardized outcomes are needed.

## Introduction

Primary molars are essential for mastication, maintenance of arch length, eruption guidance of permanent teeth, and occlusal stability during growth (1). Their preservation is clinically important because premature loss may cause space loss and compromise the developing dentition (1). Moreover, primary teeth have thinner enamel and dentin, larger pulp chambers, and pulp horns located closer to the external surface, making extensive caries progression and restorative management particularly challenging (2 , 3). When primary molars present extensive caries, multisurface involvement, or structural compromise after pulp therapy, full-coverage restorations are often indicated to provide coronal seal, functional resistance, and long-term stability (3 - 6). Stainless steel crowns (SSCs) remain the conventional reference because of their durability, clinical predictability, and favorable longevity (4 , 7). However, their metallic appearance is a major limitation in an era of increasing esthetic expectations from parents and children. Preformed zirconia crowns have emerged as an esthetic alternative to SSCs and have shown favorable outcomes in appearance, parental satisfaction, and gingival response (8 - 10). Nevertheless, their rigidity requires passive seating and may involve more aggressive tooth reduction, which is relevant in primary molars because of their limited hard-tissue thickness and pulpal proximity (2 , 3 , 8 - 10). In addition, preformed crowns are standardized and may not fully adapt to individual tooth morphology. Digital workflows, including scanning, CAD/CAM milling, and 3D printing, allow the fabrication of customized esthetic crowns adapted to the prepared primary molar (11 - 16). These restorations may improve marginal and internal adaptation and expand the use of different biomaterials, including zirconia, polymethyl methacrylate, resin-based composites, hybrid ceramics, and printed resins. However, the evidence supporting digitally fabricated custom esthetic crowns for primary molars remains limited and heterogeneous. Previous reviews have mainly focused on zirconia crowns, with less attention to customized digital crowns and comparative material performance (8 - 10). Therefore, this systematic review aimed to synthesize the available in vitro and clinical evidence on digitally fabricated custom esthetic crowns for primary molars, focusing on adaptation, mechanical behavior, antagonist wear, clinical performance, gingival health, and patient- and parent-centered outcomes.

## Material and Methods

This systematic review was conducted according to the PRISMA 2020 recommendations (17 , 18). The review question was structured according to the PICO framework as follows: "in primary molars requiring full-coverage restoration, how do digitally fabricated custom esthetic crowns compare with conventional pediatric crown systems in terms of marginal and internal adaptation, mechanical behavior, antagonist wear, clinical performance, gingival health, and patient- and parent-centered outcomes?" A literature search was performed in PubMed, Scopus, and Web of Science. The search strategy combined controlled vocabulary, when available, and free-text terms related to primary teeth, primary molars, pediatric crowns, stainless steel crowns, zirconia crowns, CAD/CAM, digital workflow, 3D printing, esthetic crowns, marginal adaptation, fracture resistance, wear, gingival health, clinical success, survival, and satisfaction. The main search was limited to studies published between 2019 and 2026. In addition, a complementary targeted search without date restriction was performed to identify relevant clinical studies not retrieved by the initial strategy. The database search was last updated in January 2026. Studies were eligible if they evaluated full-coverage esthetic crowns for primary molars fabricated through digital workflows, including subtractive CAD/CAM milling or additive manufacturing. Eligible materials included zirconia, polymethyl methacrylate, resin-based CAD/CAM composites, hybrid ceramics, lithium disilicate, PEEK, and 3D-printed resins. Randomized clinical trials, comparative clinical studies, in vitro studies, finite element analyses, and case series were considered for inclusion. Studies were excluded if they evaluated teeth other than primary molars without separate data for primary molars, assessed restorations other than full-coverage digitally fabricated crowns, focused on endocrowns, used unrelated comparators, or reported outcomes outside the scope of the review. Duplicate records and studies with insufficient extractable data were also excluded. After duplicate removal, titles and abstracts were screened, and potentially eligible studies were assessed in full text. Data were extracted on author, year, study design, restorative material, fabrication method, comparator, sample size or number of participants, follow-up period when applicable, outcomes assessed, and main findings. Risk of bias was assessed according to study design. Randomized clinical trials were evaluated using the revised Cochrane risk-of-bias tool for randomized trials, RoB 2 (19). In vitro studies were assessed using the QUIN tool for dental in vitro studies (20). The finite element analysis was evaluated narratively because no standardized risk-of-bias tool was considered directly applicable to this design. A meta-analysis was not performed because of substantial heterogeneity in study design, materials, fabrication methods, comparators, aging protocols, measurement techniques, follow-up periods, and outcome definitions. Therefore, a qualitative synthesis was performed, grouping the evidence according to study type and main outcome category. This systematic review was not prospectively registered due to the exploratory nature of the review and the heterogeneity expected across in vitro, clinical, and finite element studies. Nevertheless, the review question, eligibility criteria, search strategy, data extraction items, and risk-of-bias approach were defined before study selection and synthesis.

## Results

**Figure 1:**
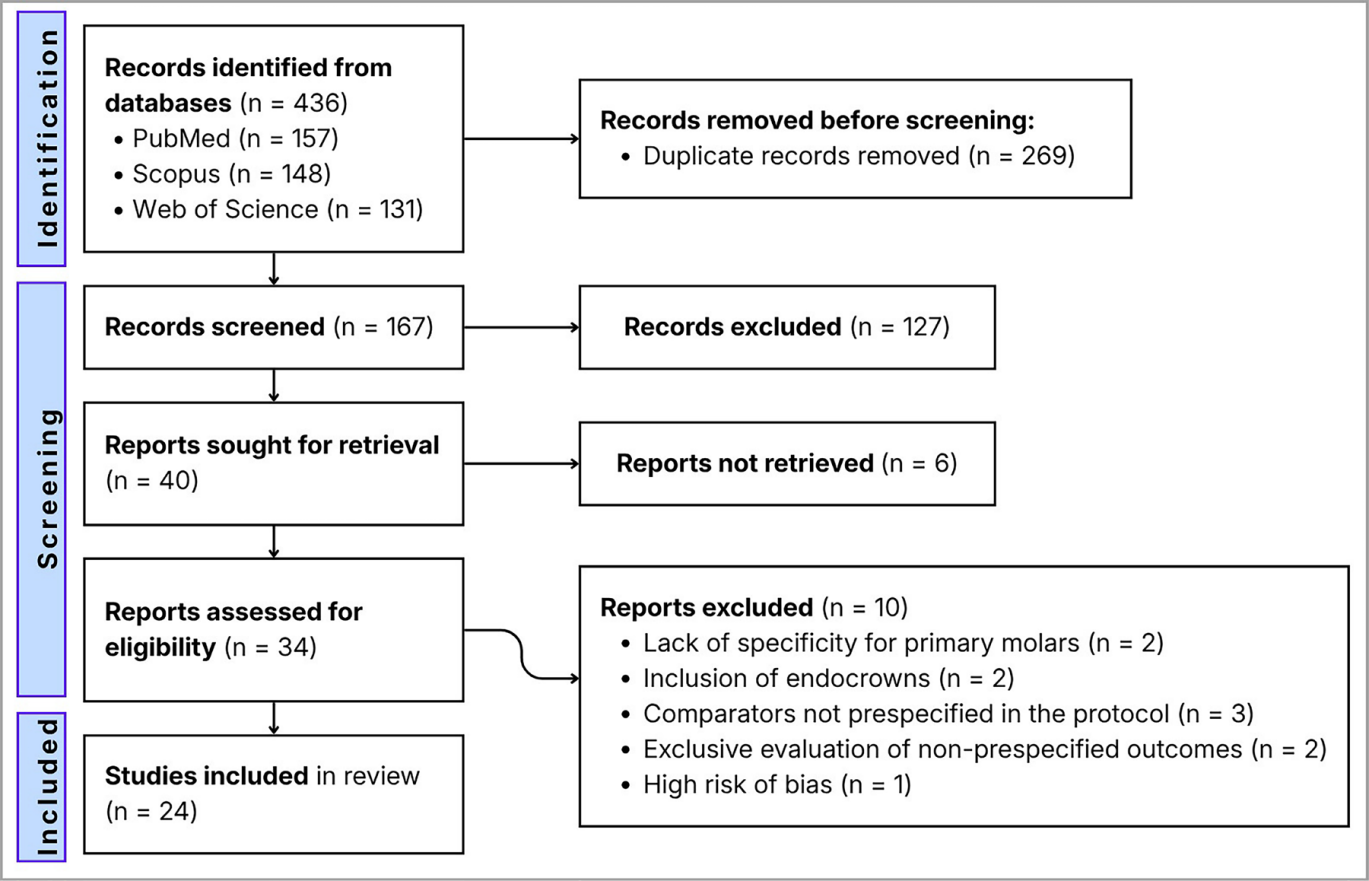
PRISMA flowchart.

1. Study selection The initial search identified 436 records through PubMed, Scopus, and Web of Science. After removal of 269 duplicates, 167 records were screened by title and abstract. Of these, 40 reports were sought for retrieval; six reports were not retrieved, and 34 reports were assessed for eligibility.. Finally, 24 studies met the eligibility criteria and were included in the qualitative synthesis. The included studies comprised 17 in vitro studies, six clinical studies, and one finite element analysis. The main reasons for exclusion at the full-text stage were absence of specific data for primary molars, evaluation of endocrowns, use of comparators outside the scope of the review, or assessment of outcomes not aligned with the predefined objectives. The study selection process was summarized using a PRISMA flow diagram (Fig. 1).

1

2. Characteristics of the included studies The included studies were published between 2019 and 2026. Most studies evaluated laboratory outcomes, whereas clinical evidence was limited to six studies with follow-up periods ranging from 9 to 36 months. The most frequently assessed materials were zirconia, 3D-printed resins, polymethyl methacrylate, CAD/CAM resin composites, hybrid ceramics, and, less frequently, PEEK. Seventeen studies evaluated CAD/CAM-milled restorations, 14 included 3D-printed resins, and 17 assessed zirconia in either prefabricated or customized form. The most common comparators were stainless steel crowns and preformed zirconia crowns. Other comparisons involved different digitally fabricated systems, including milled and printed polymer-based crowns, CAD/CAM ceramics, hybrid ceramics, and resin-matrix materials. The most frequently reported outcomes were marginal and internal adaptation, fracture resistance, fatigue behavior, antagonist wear, clinical survival or retention, marginal integrity, gingival response, and parental or child satisfaction. The main characteristics of the included studies are summarized in Table 1.

Table 1

**Figure 2:**
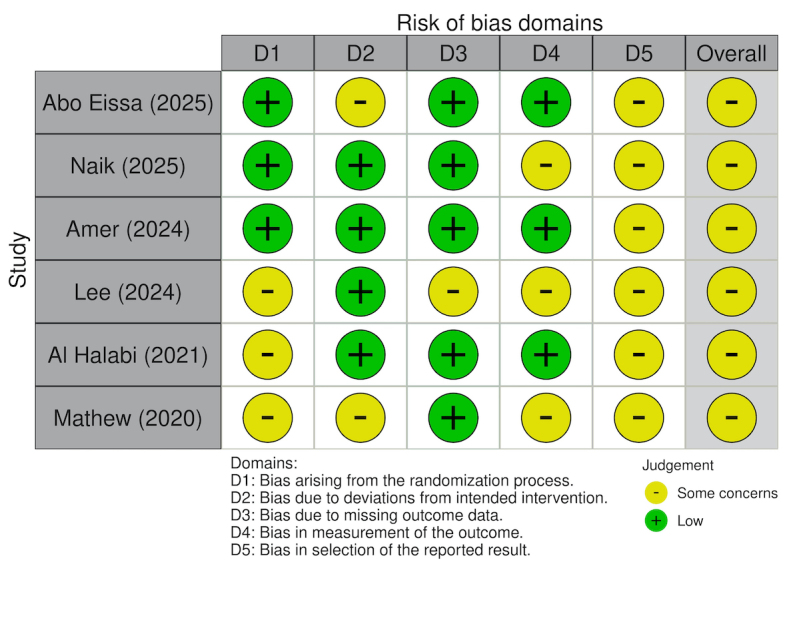
RoB 2 assessment of clinical studies.

**Figure 3:**
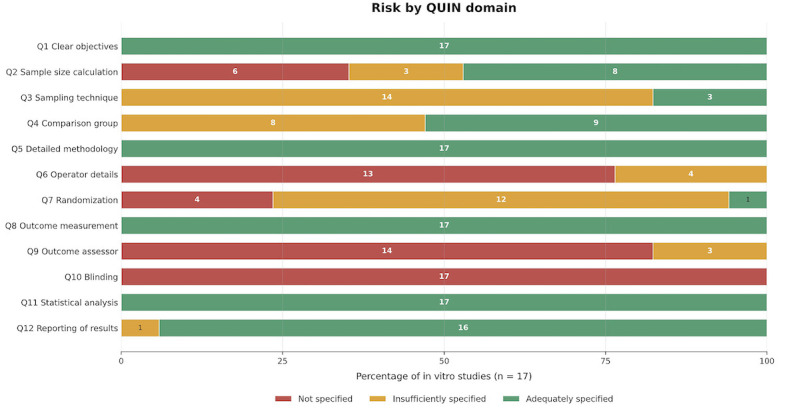
QUIN assessment of in vitro studies.

3. Marginal and internal adaptation Studies evaluating marginal and internal adaptation used heterogeneous methods, including micro-computed tomography, marginal gap measurements, and triple-scan protocols. In general, digitally customized crowns showed favorable adaptation values, although results differed according to material and fabrication method. One in vitro study reported the highest marginal and internal gaps for preformed zirconia crowns, whereas milled restorations showed the lowest values; the 3D-printed resin group showed the highest absolute marginal discrepancy (13). Another micro-CT study found the lowest marginal gap in CAD-milled crowns and the lowest axial gap in AI-designed 3D-printed crowns (21). A triple-scan evaluation reported lower gap volumes for DLP-printed crowns than for SLA-printed or milled resin-based crowns (22). However, not all studies found relevant differences between digitally fabricated materials, as one study reported no significant difference in trueness between milled zirconia and milled hybrid ceramic crowns (23). 4. Fracture resistance, fatigue behavior, and mechanical outcomes Mechanical outcomes varied substantially across studies. CAD/CAM zirconia frequently showed high fracture resistance values. Ouz et al. reported the highest fracture load for CAD/CAM zirconia crowns after artificial aging, while all composite strip crowns failed after aging (15). Elian El Hayek et al. found higher fracture strength for customized milled zirconia crowns than for preformed zirconia crowns (24). Similar findings were reported in studies comparing CAD/CAM zirconia, preformed zirconia, hybrid ceramic, and other esthetic crown materials (25 , 26). Kist et al. reported 100% survival during chewing simulation for zirconia groups and higher fracture load for CAD/CAM zirconia than for several preformed zirconia systems (27). Composite CAD/CAM crowns containing S-PRG filler also showed acceptable fatigue performance, particularly when resin cement was used (28). Polymer-based and printed materials showed variable results. Elnagar et al. reported higher fracture resistance for a microfilled hybrid 3D-printed resin crown than for preformed zirconia, with similar marginal gap values (29). Kim et al. found no significant differences in fracture resistance between two 3D-printed resin crowns and preformed zirconia crowns at 0.7 mm thickness (30). Park et al. also reported comparable mean fracture resistance between printed resin crowns and zirconia crowns, although surface changes such as microcracks and filler loss were observed after thermomechanical loading (31). In another study, fracture resistance of 3D-printed resin crowns depended on printing technology and cement type, with DLP outperforming SLA and thermocycling reducing resistance in all groups (32). 5. Antagonist wear and biomechanical behavior Wear outcomes were less uniform. Ravi et al. reported a fracture resistance gradient of zirconia, followed by PMMA and 3D-printed resin, while PMMA showed a more favorable antagonist wear profile (33). Yamanaka et al. observed lower cervical strain in zirconia-based combinations but greater antagonist enamel wear, whereas CAD/CAM composite crowns showed lower antagonist wear in the simulated deciduous tooth model (34). Akta and Bankolu Güngör reported high fracture resistance for milled PEEK and favorable 3D wear behavior for milled resin-matrix ceramic; however, no significant differences were observed in antagonist enamel wear between the tested materials (35). The finite element analysis reported differences in stress distribution between a 3D-printed resin crown and a preformed zirconia crown (36). 6. Clinical outcomes The six clinical studies evaluated stainless steel crowns, preformed zirconia crowns, CAD/CAM PMMA crowns, 3D-printed resin crowns, CAD/CAM zirconia crowns, and CAD/CAM hybrid ceramic crowns. Follow-up ranged from 9 to 36 months. Lee et al. reported 100% survival for stainless steel crowns and 82.1% survival for 3D-printed resin crowns after 12 months. The printed resin group also showed higher gingival index scores, greater occlusal wear, and clinical findings such as cracks and discoloration (37). Al-Halabi et al. found no significant difference in retention between CAD/CAM PMMA crowns and 3D-printed resin crowns at 12 months, with retention rates of 80% and 84%, respectively; gingival response was more favorable in the printed crown group (14). Naik et al. compared milled PMMA, 3D-printed photopolymer resin, and preformed zirconia crowns. At 9 months, retention rates were 80%, 93.33%, and 86.66%, respectively, with better marginal adaptation for printed resin crowns and absence of gingivitis in the polymeric groups (38). Amer and Abdellatif evaluated antagonist enamel wear in vivo and reported the following sequence of wear: CAD/CAM zirconia, preformed zirconia, CAD/CAM hybrid ceramic, and stainless steel crowns, with significant differences among groups (39). Abo-Eissa et al. found no significant differences between CAD/CAM PMMA and 3D-printed PMMA crowns regarding marginal integrity, proximal contact, gingival index, or antagonist wear. However, at 9 months, the CAD/CAM group showed a higher proportion of crowns without visible defects and better color match and parental satisfaction (40). Mathew et al. reported 100% retention for both stainless steel crowns and preformed zirconia crowns after 36 months. Differences were observed in plaque accumulation and esthetic satisfaction, with zirconia crowns showing better parental and child satisfaction related to color (41). Patient- and parent-centered outcomes were reported mainly through satisfaction or acceptance scales. No study used a formal oral-health-related quality of life instrument. 7. Risk of bias and methodological quality Among the six clinical studies, the overall RoB 2 judgment was "some concerns" in all cases. The main limitations were incomplete reporting of randomization and allocation concealment, absence of blinded outcome assessment in some trials, and insufficiently detailed analysis plans. Among the 17 in vitro studies, QUIN assessment showed a predominance of medium risk of bias, with four studies classified as low risk and the remaining studies as medium risk. The most frequent methodological limitations were absence of formal sample size calculation, insufficient description of sampling procedures, limited reporting of operators and outcome assessors, and lack of blinding. The finite element analysis was evaluated narratively and was considered to have moderate methodological credibility and limited clinical transferability. The RoB 2 assessment of the included clinical studies and the QUIN assessment of the included in vitro studies are presented in Figures 2 and 3, respectively.

2

3

## Discussion

This systematic review found that digitally fabricated custom esthetic crowns are a promising restorative option for primary molars, but the current evidence does not support their consistent superiority over stainless steel crowns. Most available studies were in vitro, while clinical evidence was limited, heterogeneous, and mainly restricted to short-term follow-up. Therefore, these restorations should be interpreted as an emerging alternative rather than a replacement for conventional pediatric crowns. The laboratory evidence suggests that customized digital crowns may provide favorable marginal and internal adaptation, particularly because CAD/CAM and 3D printing allow the restoration to be designed according to the morphology of the prepared primary molar (13 , 21 , 22). This potential advantage is clinically relevant, since inadequate adaptation may compromise cement stability, plaque control, and restoration longevity. However, the studies used different measurement methods, materials, and protocols, which limits direct comparison. Mechanical performance was also material-dependent. CAD/CAM zirconia frequently showed high fracture resistance and favorable behavior after simulated aging (15 , 24 - 27). Nevertheless, fracture resistance alone should not be considered equivalent to clinical superiority. In primary molars, the restorative material must also be evaluated in relation to tooth reduction, pulpal proximity, cementation, gingival response, and antagonist wear. This is especially important because primary teeth have thinner hard tissues and larger pulp chambers than permanent teeth (2 , 3). Polymeric and resin-based digital crowns showed more variable results. Some studies reported acceptable fracture resistance, adaptation, and short-term clinical performance for 3D-printed resins and CAD/CAM PMMA crowns (14 , 29 - 32 , 38 , 40). However, other studies described disadvantages such as discoloration, cracks, occlusal wear, visible defects, or lower survival compared with stainless steel crowns (37 , 40). These findings suggest that their performance depends strongly on material composition, fabrication method, post-processing, cementation, and aging conditions. Clinical studies showed acceptable short-term outcomes for several esthetic digital crowns, but stainless steel crowns remained a highly predictable comparator (37 , 41). Zirconia crowns showed clear esthetic advantages and high parental or child satisfaction (38 , 41), although greater antagonist wear was reported in some comparisons (39). Patient-centered outcomes were generally limited to satisfaction or acceptance scales, and no study used validated oral-health-related quality-of-life instruments. Despite the results, this review has some limitations. First, the review protocol was not prospectively registered. However, the review question, eligibility criteria, search strategy, data extraction items, and risk-of-bias approach were defined before study selection and synthesis. The heterogeneity of study designs, materials, comparators, follow-up periods, and outcome definitions prevented meta-analysis. In addition, most evidence was preclinical, the number of clinical studies was small, and most clinical follow-ups were short. Some materials, such as PEEK, hybrid ceramics, and CAD/CAM composites, were represented by few studies, limiting comparative conclusions. Overall, digitally fabricated custom esthetic crowns may offer advantages in esthetics, individualization, and adaptation, but current evidence remains insufficient to establish them as superior to conventional crowns. Further randomized clinical trials with larger samples, longer follow-up, standardized outcomes, and validated patient-centered measures are needed to define their clinical role in pediatric restorative dentistry.

## Conclusions

Current clinical evidence remains limited and mostly short-term, and does not demonstrate consistent superiority over stainless steel crowns, which remain a predictable comparator. Therefore, digitally fabricated custom esthetic crowns should not yet be considered a definitive replacement for conventional pediatric crowns. Further well-designed randomized clinical trials with larger samples, longer follow-up, standardized outcomes, and validated patient-centered measures are needed to define their clinical role in pediatric restorative dentistry.

## Figures and Tables

**Table 1:** Characteristics of the included studies.

Author (Year)	Study Design	Restorative Material(s)	Sample Size	Primary Outcome
Kist (2019)	In vitro study	Three prefabricated zirconia systems, CAD/CAM zirconia, veneered stainless steel crowns (SSCs), and conventional SSCs	72 crowns	Fracture load and survival after chewing simulation
Mathew (2020)	Comparative clinical study	Stainless steel crowns (SSCs) versus prefabricated zirconia crowns	60 primary molars (30 per group)	Clinical success, plaque accumulation, and parent/child satisfaction
Al-Halabi (2021)	Randomized clinical trial	Indirect CAD/CAM PMMA crowns versus 3D-printed resin crowns	50 primary molars (25 per group)	Clinical performance and retention at 12 months
Oğuz (2022)	In vitro study	Prefabricated fiberglass crowns, CAD/CAM zirconia crowns, CAD/CAM resin-ceramic crowns, composite strip crowns, and prefabricated zirconia crowns	50 crowns (10 per group)	Fracture resistance after artificial aging
Elian El Hayek (2022)	In vitro study	Prefabricated zirconia crowns versus customized/milled zirconia crowns	30 primary molars (15 per group)	Fracture resistance
Kim (2022)	In vitro study	3D-printed resins (Graphy TC-80DP and NextDent C&B MFH) versus prefabricated zirconia crowns	45 crowns for fracture testing and 44 discs for biaxial flexural testing	Fracture resistance, biaxial flexural strength, and dynamic mechanical behavior
Nakase (2023)	In vitro study	Experimental CAD/CAM composite with S-PRG filler versus commercial CAD/CAM composite	68 crown specimens, plus bond strength testing on primary teeth	Fatigue durability and reliability
Park (2023)	In vitro study	3D-printed resin crowns with different resins and thicknesses versus zirconia control	100 crowns, plus 50 antagonists for thermomechanical loading	Fracture load and surface characteristics
El Hayek (2024a)	In vitro study	Milled zirconia crowns versus milled hybrid ceramic crowns	28 crowns (14 per group)	Trueness/accuracy relative to the digital design file
Lee (2024)	Randomized clinical trial	3D-printed resin crowns versus stainless steel crowns (SSCs)	56 primary molars (28 per group)	Survival and clinical performance
Aktaş (2024a)	In vitro study	Permanent 3D-printed resin crowns fabricated by DLP or SLA using different cements	96 crowns	Fracture resistance according to printing technology and cement type
El Hayek (2024b)	In vitro study	Prefabricated zirconia, CAD/CAM zirconia, CAD/CAM ceramic, and CAD/CAM hybrid composite crowns	60 primary molars (15 per group)	Fracture resistance
Aktaş (2024b)	In vitro study	Prefabricated zirconia, prefabricated composite, milled composite, milled ceramic-matrix resin, milled PEEK, and 3D-printed resin	60 specimens (6 groups, 10 per group)	Antagonist enamel wear and fracture resistance
Amer (2024)	Randomized clinical trial	Stainless steel crowns (SSCs), prefabricated zirconia, CAD/CAM zirconia, and CAD/CAM hybrid ceramic crowns	40 primary molars (10 per group)	In vivo antagonist enamel wear
Aktaş (2024c)	In vitro study	Milled and 3D-printed esthetic resin crowns designed using CAD software or artificial intelligence	40 crowns (10 per group)	Marginal and internal adaptation assessed by micro-CT
Aktaş (2025a)	In vitro study	3D-printed crowns, milled ceramic-matrix resin crowns, and prefabricated zirconia crowns	30 crowns (10 per group)	Marginal and internal adaptation and absolute marginal discrepancy
Elnagar (2025)	In vitro study	3D-printed microfilled hybrid resin crowns versus prefabricated zirconia crowns	20 primary molars (10 per group)	Fracture resistance and marginal gap
Ravi (2025)	In vitro study	Zirconia, PMMA, and 3D-printed photopolymerizable resin crowns	30 crowns (10 per group), plus 15 antagonist teeth	Fracture resistance and antagonist occlusal wear
Elkhodary (2025)	In vitro study	Veneered metal crowns, prefabricated zirconia crowns, CAD/CAM zirconia crowns, and CAD/CAM hybrid ceramic crowns	40 primary molars (10 per group)	Marginal gap distance and dynamic fatigue performance
Kalaskar (2025)	Finite element analysis	3D-printed NextDent resin crown versus prefabricated zirconia crown	2 finite element models	Stress and strain distribution
Aktaş (2025b)	In vitro study	DLP-printed resin crowns, SLA-printed resin crowns, and milled composite block crowns	30 crowns (10 per group)	Marginal and internal fit assessed by the triple-scan protocol
Abo-Eissa (2025)	Split-mouth randomized clinical trial	CAD/CAM PMMA crowns versus 3D-printed PMMA crowns	30 primary molars in 15 children (15 per group)	Clinical performance according to modified USPHS criteria
Naik (2025)	Randomized clinical trial	CAD/CAM milled PMMA crowns versus 3D-printed photopolymerizable resin crowns versus prefabricated zirconia crowns	45 primary molars (15 per group)	Retention, clinical performance according to USPHS criteria, marginal adaptation, and gingival health
Yamanaka (2026)	In vitro biomechanical simulated model	Zirconia, stainless steel crowns (SSCs), and CAD/CAM composite crowns combined with different core materials	48 specimens for strain analysis, plus 24 antagonists for wear testing	Cervical strain and antagonist enamel wear

1

## Data Availability

The datasets used and/or analyzed during the current study are available from the corresponding author.
